# Efficiency Quality Index (EQI)—Implementing a Novel Metric That Delivers Overall Institutional Excellence and Value for Patients

**DOI:** 10.3389/fsurg.2020.604916

**Published:** 2021-02-01

**Authors:** Robert J. Cerfolio, Stephanie H. Chang

**Affiliations:** Department of Cardiothoracic Surgery, New York University Langone Health, New York, NY, United States

**Keywords:** quality, metrics, outcomes—health care, surgery, value, thoracic surgery

## Abstract

In the last decade, healthcare systems have shifted their focus from increased volume of patients and procedures to improving patient outcomes and quality. While there are many societies and companies that have surrogate measures of excellence, these metrics are determined by those who do not directly participate and fully understand the best measurements of quality. In order to better assess quality and value, the Efficiency Quality Index (EQI) was created. The novel aspect of the EQI is the determination of metrics by the physicians who actually perform the procedures, in order to create an accurate and fair measurement of performance and outcomes. In this article, we describe how to create and implement the EQI, as well as outline its benefits.

## Introduction

Over the past several years, there has been a movement toward quality over volume in the care of patients, with greater emphasis now placed on delivering the highest quality of care as opposed to achieving the highest volume (1). Unfortunately, evaluating healthcare is similar to being a food critic—the arbitrator of what is quality is highly subjective and difficult to objectify. To address this problem, over the past several years, many have tried to quantify quality.

Insurance companies value quality, but the reality is most private payers look for the lowest cost and do not fully understand episodes of care. Consumers have always focused on outcomes but find it impossible to decipher paid advertisements and branding from what is actually better care from competitors. In response, there has been an explosion of both non-profit and for-profit companies and societies dedicated to the housing, collection, and interpretation of data. The “alphabet soup” of rankings, such as the National Surgical Quality Improvement Program (NSQIP), the society of thoracic surgery (STS), Vizient, the United States News and World Report (USNW Report), Centers for Medicare and Medicaid Services (CMS) Star Rating, Leapfrog, and National healthcare safety network (NSHN), attempt but often fall short of capturing the true, detailed measures of quality for specialties, such as the rate of R0 resection, quality of lymphadenectomy, and blood loss in lung resections for lung cancer.

Vizient appears to be the best arbitrator because they only use objective metrics of outcomes and do NOT use reputation factor (2). However, physicians who deliver care to patients have a better understanding of the true indicators of quality care of and for patients. In response to the opaque quality-ranking-industrial-complex and long-time need for improved measurements (3), we have created and refined a more detailed system for our services and physicians to evaluate their performance: the Efficiency Quality Index (EQI). This novel metric engages staff because it is fair and transparent; allows the participants to ensure the data are correct and to decide what is the best metrics of quality in their specific field or operation; permits participants to remove data if others they are compared to agree; ensures an accurate attribution of complications and quality; and create evolving iterations and metrics as frequently as needed to best serve patients.

## Creating an EQI

Most professionals, not just in healthcare, perform repeated tasks, with the expectation of constant improvement over time. Those who carry out the job on a daily basis are better judges of the quality of the task, compared to a remote, myopic arbitrator who may be incentivized by commercial interest or, even worse, blinded by a reputation that often is historic. The EQI is a composite score of the key measures of performance. Not only do the performers determine the metrics of the quality, but they also assign its numerical value and weight. It is a scoring system created by the players of the game themselves. Importantly each participant is allowed to ensure that every data point assigned to him or her is accurate. If one is not, they should question it, but only the entire group is able to change or remove a data point after public vetting. This is critical for engagement, fairness, and transparency.

To build an EQI at our institution, one must follow these steps listed below:

1. The division chief, with input from their physicians, select EQI procedures and all metrics to use
2. The analytics team creates a database with relevant data—where data points do not exist, divisions themselves manually audit charts
3. Division chiefs submit subjective rankings to “gut check” the system
4. The analytics team provides the division chief with the score, and the division chief shares all cases and outcomes with their individual physicians for vetting
5. Data or attribution errors are fixed—coding issues are caught and resolved
6. Divisions review together, as a team, to identify opportunities and share best practice according to top performers.

The metrics we ask leaders to pick do not just evaluate quality; that is only one piece of the equation, since Value = Quality/Cost. When comparing a person who can clean an operating room in 15 min just as well as a person who takes 30 min, they are not equal in value. To best push our teams to improve performance, we must show that individuals can achieve the same outcomes at different levels of efficiency. Therefore, any measure of quality is incomplete if it does not measure the time it takes to perform that task. We have to consider quality as judged by a unit of time or effort, in order to really measure value. Only when we are transparent can we inspire others to succeed at a higher level. Just as the sub-4-min mile was once unimaginable and is now the goal for competitive runners, seeing what is possible regarding efficiency leads to the elevation of all performance. This is especially true if wins are celebrated and losses are only seen as opportunities to get better. Less experienced physicians must be encouraged and shown how to improve performance via positive coaching. This culture is critical for the EQI to appropriately function in any workplace. The culture that surrounds it must be a positive one that encourages healthy friendly competition, with less efficient performers viewing each evaluation as a chance to improve and better serve patients.

## Example of EQI

Our current EQI for lobectomy evaluates the following components, with the weight of each included in parentheses.

° OR time incision to close (10)
° Total OR time (10)
° Observed to expected length of stay (18)
° Case mix index adjusted cost (10)
° Early discharge rate (10)
° Readmissions (12)
° Hospital-acquired conditions (20)
° Surgical site infection (14)
° 30-day mortality (25)
° 90-day mortality (20)
° Hospital Consumer Assessment of Healthcare Providers and Systems score (15)
° OR tranfusions (10)
° Conversion to thoracotomy (15)
° Number of lymph nodes per case (15)
° Total nodal stations per care (20)
° Pneumonia requiring trach (10)
° Return to OR for bleeding (10)

The data are collected for all surgeons, and the scores are normalized between the surgeons to come up with a score for each section, with the EQI as the total composite score (see Table 1).

Table 1Example of lobectomy EQI.

**Table:** 

**Provider**	**Total OR min/case**	**Pts (10)**	**Cut-Close min/case**	**Pts (10)**	**O/E LOS**	**Pts (18)**	**CMI adj cost/case**	**Pts (10)**	**Early discharge rate**	**Pts (10)**	**Readmit rate**	**Pts (12)**	**HAC rate**	**Pts (20)**	**SSI rate**	**Pts (14)**	**30-day mortality**	**Pts (25)**	**90-day mortality**	**Pts (20)**
1	159	10	110	10	0.57	17	$5,700	3	75%	7	10%	4	0%	20	0%	14	0%	25	0%	20
2	197	7	136	8	0.56	18	$4,930	10	68%	4	4%	9	0%	20	0%	14	0%	25	0%	20
3	206	7	133	8	0.62	16	$6,000	0	57%	0	7%	7	0%	20	0%	14	0%	25	0%	20
4	298	0	216	0	1.04	0	$6,050	0	65%	3	12%	0	0%	20	0%	14	0%	25	8%	0

**Table:** 

**Provider**	**HCAHPs score**	**Pts (15)**	**OR transfusions**	**Pts (10)**	**Convert to Open**	**Pts (15)**	**Lymph node number/case**	**Pts (15)**	**Lymph node station/case**	**Pts (20)**	**Pneumonia requiring trach**	**Pts (10)**	**Return to OR for bleed**	**Pts (10)**	**Total EQI (244)**
1	2.9	15	0	10	0	15	27	15	7.2	20	0	10	0	10	225
2	2.8	11	0	10	0	15	14	10	4.7	7	0	10	0	10	208
3	2.7	5	0	10	1	8	29	15	5	13	0	10	0	10	187
4	2.55	0	0	10	2	0	11	0	4	7	0	10	0	10	103

*CMI adj cost, case mix index adjusted cost; HAC, hospital-acquired condition; Min, minutes; OR, operating room; O/E LOS, observed-to-expected length of stay; Pts, points; SSI, surgical site infection*.

*EQI, efficiency quality index; HCAHPs, hospital consumer assessment of healthcare providers and systems scores; OR, operating room; Pts, points*.

## Utilizing EQI

We invented the EQI nearly 10 years ago as thoracic surgeons. My partners and I have revamped it many times over the years. Table 1 shows the current measurements of quality metrics for pulmonary lobectomy and segmentectomy that we use at New York University (NYU) Langone Health for patients with non-small cell lung cancer. This is the seventh iteration for the quality metrics on the EQI for lung resection. In addition, we measure non-procedure-specific metrics of quality that are shared hospital metrics (such as 30-day mortality, surgical site infections, procedure time, etc.). In combination, performance on these metrics is weighted and combined into a normalized composite score that ranks physicians in each domain, and overall.

Several quality metrics are used to combat against gaming the system. First, we have the resident and/or fellow report teaching scores to ensure that we are not just efficient at the price of education. For those that argue that they teach best when quadrupling the length of a procedure, this provides neither the patient with quality care nor teaches the resident about surgical efficiency. Second, we measure re-admission rates and patient satisfaction scores to ensure that we are not rushing unfit patients out of the hospital.

Over the last few years as a Chief Operating Officer of a hospital and of a healthcare system, we have applied the EQI across many services at NYU Langone. Additionally, the EQI extends beyond physicians. It has also been used in non-clinical services throughout the hospital, including building services, food and nutrition, and transport services.

A cornerstone of EQI is radical transparency. EQI scores must be easy to understand and reviewed interactively. Learning from others is an important way to improve. In this endeavor, humility is critical. We do not know who is the best in one specific procedure or episode of care until we measure it. We must all be willing to admit where we can improve. We all want to be the best, but, when we are not, we must learn from those who are.

## Benefits of EQI

There were several original goals of EQI:

1. To eliminate or mitigate the inherent subjectivity each individual applies to their own work.
2. To eliminate the fact that we all think we are the best or in the top 10% in quality.
3. To engage staff in rankings of quality in order to better deliver higher quality care to patients.
4. To eliminate some physicians from performing specific procedures that others do better.
5. To eliminate the ego from these difficult conversations by using objective accurate data that the individuals themselves have approved and chosen as the best metrics of quality.
6. To eliminate the first two complaints that every MD offers when confronted with their own poor quality data:
  - “The data is wrong”: This is not possible with the EQI because it is data that they vetted and submitted themselves.
  - “The metrics of quality are inaccurate”: This argument is moot with the EQI, as the physician helped determine the metrics.

Through objective data, we can push ourselves to improve by discretely measuring what makes “best care” the best. As we have evolved, we have learned the following lessons: The greatest impact occurs when the participants get together every 3–6 months to iterate and tweak the system, in order to provide better measurements of efficiency and quality, and to ensure the data are 100% accurate. For thoracic surgery, this process led to 7 versions in 10 years. The original version vs. the current EQI for lobectomy/segmentectomy included the following metrics:

- Original
  ° OR time (incision to close)
  ° Cost per case
  ° First case on time starts
- Current:
  ° OR time (incision to close AND total)
  ° Observed-to-expected length of stay
  ° Case mix index adjusted cost
  ° Early discharge rate
  ° Readmissions
  ° Hospital-acquired conditions
  ° Surgical site infection
  ° 30- and 90-day mortality
  ° Hospital Consumer Assessment of Healthcare Providers and Systems score
  ° OR tranfusions
  ° Conversion to thoracotomy
  ° Number of lymph nodes per case
  ° Total nodal stations per care
  ° Pneumonia requiring trach
  ° Return to OR for bleeding

For coronary artery bypass graft procedural metrics, it meant three versions in 9 months. Process change should relate back to the metrics in the EQI—if we think something is important, it can best be improved if we can measure it, track it, and then work to improve it.

## Results of EQI

We have been using the EQI for the past several years at NYU, and the results are staggering when looking at national scoring systems. While a perfect arbitrator for quality does not exist, the best national scoring systems are the STS and Vizient. More commonly used ranking systems, such as USNW Report and Leapfrog, were also examined as an objective way of evaluating quality before and after the implementation of the EQI. Thoracic surgery has achieved a three-star STS ranking for the first time ever. A three-star STS ranking has only been given to 6 out of 212 hospitals in North America. In addition, our Vizient hospital ranking has been either first or second over the past several years. Our score ratings are shown in Table 2. Within the thoracic division, the EQI score discussed at quarterly meetings have led to a 10% increase in the number of lymph nodes per case, improved observed-to-expected length of stay (from 0.57 to 0.41 for one surgeon), and a $1000 decrease in cost per case.

**Table 2:** New York University quality ratings by year.

**Year**	**Leapfrog**	**USNWR^a^ best hospitals**	**USNWR^a^ best medical schools**	**CMS^b^ star rating**	**Vizient**
2017	C	#19	#11	5/5 stars	#2
2018	C	#15	#12	5/5 stars	#3
2019	A	#9	#3	4/5 stars	#2
2020	Pending	#9	#9	5/5 stars	Pending
2021	Pending	Pending	#4	Pending	Pending

a *USNWR, United States news and world report*;

b *CMS, centers for medicare and medicaid services*.

## Conclusion

As experts in our field, we must be willing to put the time in to create internal measures needed to improve. The intersection of quality and efficiency is where we must focus to best serve our mission and patients. At our hospital, for this reason, we believe the EQI is the single most important metric to provide patients with better care, healthcare systems with greater value, and physicians, nurses, and staff members in healthcare with the information they need to improve.

We should not allow outside arbitrators to be the determinants of quality, especially if they do not fully understand the best measurements of quality. If we do not actively intervene, we then aim for targets that do not lead to improved patient care. The EQI allows those who perform the tasks to be involved directly in the process of setting performance targets. We vet the data to ensure that they are accurate and that the attribution of complications and success is fair. Aligning EQI with the realities of external pressures (CMS penalties, for example) is of critical benefit to any healthcare system, but what cannot be lost is the urgency for us to be the active agents and participants in the measurement of our own value. Engagement in the process is ONLY possible with a positive collaborative culture and, through this culture shift, we can influence real change. We must be the owners of quality assessment to avoid becoming passive consumers of sub-optimal measures.

## Author Contributions

RC was responsible for all aspects of the paper, including design, writing, and editing. All authors contributed to the article and approved the submitted version.

## Conflict of Interest

The authors declare that the research was conducted in the absence of any commercial or financial relationships that could be construed as a potential conflict of interest.
